# Mechanisms and targeted prevention of hepatic osteodystrophy caused by a low concentration of di-(2-ethylhexyl)-phthalate

**DOI:** 10.3389/fimmu.2025.1552150

**Published:** 2025-03-10

**Authors:** Qinming Hui, Xinru Du, Maoxuan Li, Sha Liu, Zhendong Wang, Sisi Song, Yancheng Gao, Ye Yang, Chunxiao Zhou, Yuan Li

**Affiliations:** ^1^ Department of Gastroenterology, The Affiliated Suzhou Hospital of Nanjing Medical University, Suzhou, China; ^2^ The Key Laboratory of Modern Toxicology, Ministry of Education, School of Public Health, Nanjing Medical University, Nanjing, China

**Keywords:** hepatic osteodystrophy, di-(2-ethylhexyl) phthalate, 14-3-3η/nuclear factor κB feedback loop, secretory proteome, targeted intervention

## Abstract

**Objectives:**

Hepatic osteodystrophy (HOD) is an important public health issue that severely affects human health. The pathogenesis of HOD is complex, and exposure to environmental pollutants plays an important role. Di-(2-ethylhexyl) phthalate (DEHP) is a persistent environmental endocrine toxicant that is present in many products, and the liver is an important target organ for its toxic effects. Our research aimed to investigate the effects of DEHP on HOD, and to reveal the underlying mechanisms and the potential key preventive approaches.

**Methods:**

The daily intake EDI of DEHP and bone density indicators for men and women from 2009 to 2018 were screened and organized from the NHANES database to reveal the population correlation between EDI and BMD; C57BL/6 female and male mice were selected to construct an animal model of DEHP induced HOD, exploring the fuchtions and mechanisms of DEHP on osteoporosis; the novel small molecule inhibitor imICA was used to inhibit the process of DEHP induced osteoporosis, further exploring the targeted inhibition pathway of DEHP induced HOD.

**Results:**

Male and female populations were exposed to a relatively lower concentration of DEHP, and that only the male population exhibited a negative correlation between DEHP exposure and bone mineral density. An in vivo study confirmed that a low dose of DEHP caused liver lesions, disrupted liver function, and induced osteoporosis in male but not female C57BL/6J mice. Regarding the molecular mechanisms, a low dose of DEHP activated the hepatic 14-3-3η/nuclear factor κB (NF-κB) positive feedback loop, which in turn modified the secretory proteome associated with bone differentiation, leading to HOD. Finally, we revealed that targeting the 14-3-3η/ NF-κB feedback loop using our novel 14-3-3η inhibitor (imICA) could prevent DEHP-induced HOD.

**Conclusion:**

A low dose of DEHP activated the hepatic 14-3-3η/ NF-κB positive feedback loop, which in turn modified the secretory proteome associated with bone differentiation and elevated IL-6 and CXCL1 levels, leading to HOD. Targeted 14-3-3η/NF-κB feedback loop using our novel 14-3-3η inhibitor, imICA, prevented DEHP-induced HOD.

## Introduction

Osteoporosis is a systemic bone disease characterized by a reduction in bone mass and mineral density as well as macro- and microstructural alterations in bone tissue. These changes result in increased bone fragility and fracture susceptibility, which significantly affects the quality of life of patients (1). Hepatic osteodystrophy (HOD) is a disorder of altered bone metabolism that occurs in patients with chronic liver disease, and is characterized by osteoporosis, bone loss, and osteochondrosis (2). Epidemiological studies have demonstrated that osteoporosis is a prevalent complication in patients with chronic liver disease, and nearly 75% of these patients with chronic liver disease typically exhibit varying degrees of bone metabolism alterations with the progression of their hepatic disease (3, 4). HOD is associated with an increased risk of refractory fractures and has a detrimental effect on long-term patient prognosis (5). Therefore, HOD have become an important public health issue that seriously affects people’s health.

The pathogeny of HOD is complex: alcoholic liver disease, non-alcoholic steatohepatopathy, primary cholestatic hepatopathy, and viral hepatitis are the classic factors (6). In recent years, the role and mechanism of hepatotoxin exposure in the initiation and development of HOD have received increasing attention (7, 8). Phthalate esters (PAEs) are the main components of plastic additives (plasticizers and softeners) that are widely used (9). Di-(2-ethylhexyl) phthalate (DEHP) is the most common PAEs, accounting for approximately 50% of the plasticizer in polyvinyl chloride (10). Owing to the non-covalent bond to plastic components, DEHP can be easily released into the environment and exposed to humans (11). When entering the liver, DEHP is hydrolyzed to mono-(2-ethylhexyl) phthalate (MEHP), which then rapidly degrades into mono-(2-ethyl-5-hydroxyhexyl) phthalate (MEHHP), mono-(2-ethyl-5-oxohexyl) phthalate (MEOHP), and mono-(2-ethyl-5-carboxypentyl) phthalate (MECPP), which are not only important biomarkers that reflect DEHP exposure but also play important biological roles, affecting liver function (12–14). Therefore, the liver is an important target organ DEHP toxicity.

Epidemiological and toxicological studies have indicated that DEHP exposure affects bone metabolism and promotes osteoporosis (15, 16). Nevertheless, if these effects caused by DEHP are induced by HOD, the potential key molecular mechanism and how to carry out precise prevention/control remain largely uninvestigated. Thus, this study intends to further explore the molecular mechanisms of DEHP exposure-induced osteoporosis (with an emphasis on HOD) in population and mouse models, and to investigate novel potential targeted intervention approaches.

## Materials and methods

### Estimation of DEHP exposure in population

Phthalate metabolite concentrations in the urine samples were obtained from the National Health and Nutrition Examination Survey (NHANES). For the current analysis, we used data in last 10 years (from 2009 to 2018). To restrict the population, we limited the participants to those with available concentration data of urinary DEHP metabolites and bone mineral density (BMD, male, 1.12 ± 0.03, n = 2171; female, 1.01 ± 0.02, n = 2077). Based on the concentrations of phthalate metabolites in urine and the classic exposure estimation model, we estimated the total daily intake (EDI) of DEHP (μg/kg·bw/day) (17).


$$\text{EDI = CV}\times \frac{M1}{M2}\times \frac{1}{\text{W}}\times \frac{1}{\text{f}}$$

where C is the urinary phthalate metabolite concentration measured in urine samples (ng/ml), V, the volume of daily urine excreted (L/day), we assumed a volume of 2.0 L for men and 1.7 L for women; M1 and M2, the respective molecular weights of parent phthalate and its metabolite (g/mol), W, body weight (kg), we took 75 kg for men while 60 kg for women; and f, the molar fraction of the urinary monoester metabolite excreted in relation to the ingested amount of phthalate, the f were 0.059, 0.23, 0.15, 0.185 for MEHP, MEHHP, MEOHP and MECPP, respectively (18). For the EDI of DEHP, we summed the EDI of the abovementioned four metabolites.

### Animals and *in vivo* treatment

All animal protocols and experimental procedures were approved by the Nanjing Medical University Animal Care and Use Committee (permit No. IACUC-2209058). Specific pathogen-free C57BL/6 male and female mice, aged 6–8 weeks, were purchased from the Nanjing Medical University Animal Center. All mice were housed indicidually under standard 12:12 light/dark conditions, at an ambient temperature of 25°C and fed *ad libitum*. DEHP (C_24_H_38_O_4_, >99.0% purity) and corn oil were purchased from MedChemExpress (Shanghai, China). The novel 14-3-3η inhibitor, 6-isopropyl-3-(((3-methoxybenzyl) amino)methyl)-1-(4-methylbenzyl)-1H-indole-2-carboxylic acid (C_29_H_33_N_2_O_3_, imICA), was modified and synthesized in our previous study. This chemical demonstrated very low hepatotoxicity; however, it exhibited an excellent targeted inhibitory effect on the 14-3-3η protein and its downstream signal transductions (19, 20). The mice were separated into NC- and DEHP-treated groups in the presence or absence of imICA (n = 5). The dosage and frequency of medication administered is 0.5 mg/kg of DEHP (gavage, daily), 5.0 mg/kg·BW (dissolved in 10% DMSO) of imICA (gavage, daily). After 12 weeks, all the mice were euthanized by inhalation of carbon dioxide. They were actively exposed to 100% CO_2_ at a replacement rate (VDR/min) between 30% and 70% for 2 min, followed by a minimum passive exposure of 3 min to ensure that the mice did not wake up during the passive exposure time. Further examination of serum and liver tissues was conducted after cervical dislocation.

### Liver pathology and liver function

Liver tissues fixed with 4% paraformaldehyde were dehydrated gradually with ethanol, and paraffin-embedded liver tissue was cut into 4-mm sections with an Ultra-Thin Microtome before hematoxylin and eosin (H&E), Sirius Red, and Masson staining. Images were captured using a panoramic-scan digital slice-scanning system (3DHISTECH Co. Ltd., Budapest, Hungary). Photomicrographs from five random fields of view using a ×10 objective were taken from each section, and the ratio of positive areas to the total area was measured using ImageJ software, as described previously (20). For the detection of liver function, blood was collected from the abdominal aorta of the mice and centrifuged at 3,000*g* for 10 min to collect serum within 1 h. Levels of alanine aminotransferase (ALT), aspartate aminotransferase (AST), total cholesterol (CHOL), triglyceride (TG), high-density lipoprotein (HDL), low-density lipoprotein (LDL), blood urea nitrogen (BUN), and CREA were analyzed using a 7100 automatic biochemical analyzer (Hitachi, Japan).

### Micro-computed tomography analysis

Following euthanasia, the femurs of both legs were fixed in 4% formalin solution for 24 h, after which they were cleaned with PBS and subsequently scanned. Micro-CT analysis was performed using a micro-CT scanner (SkyScan; Bruker, Germany) with a resolution of 9 μm/pixel. Once the scanning process was complete, the original image orientation of the femur was corrected using the Data-Viewer software (Bruker). Subsequently, all scans were rotated by 180° to correct for the reconstruction. The images obtained following correction were selected manually for three-dimensional reconstruction and analysis of the region of interest (ROI) in the vicinity of the distal femur and growth plate using the CTAn 1.10 software (Bruker). The metrics included trabecular thickness (Tb.Th; mm), trabecular number (Tb.N; 1/mm), and structural pattern factor (SMI).

### Cytokine antibody array and bioinformatics analysis

Cytokines were detected using Mouse Inflammation Array G1 (AAM-INF-G1) manufactured by RayBio (Guangzhou, China) according to the manufacturer’s instructions. Mouse liver tissues were lysed with cold RIPA lysis buffer (Beyotime), and protein concentrations were measured using the bicinchoninic acid assay kit (BCA, Beyotime). After blocking the array chip, 100 µl of the sample (tissue protein) was added to each sub-array for incubation. After overnight incubation at 4°C, the glass chip was cleaned using a Thermo Scientific Wellwash Versa Chip Washer. Subsequently, the glass chip was incubated with biotin-conjugated antibodies and then with fluorescent dye-conjugated streptavidin. An InnoScan 300 Microarray Scanner (wavelength, 532 nm; resolution, 10 µm; Innopsys, France) was used to measure the fluorescence, GenePix Pro 6.0 software (Axon, USA), was used to extract the data, and Cytokine Antibody Array software (RayBio) was used to analyze the data. Gene Ontology (GO) and Kyoto Encylopedia of Genes and Genomes (KEGG) enrichment analysis were conducted to reveal the biological functions and characteristics via the Database for Annotation, Visualization and Integrated Discovery database (DAVID).

### Quantitative real-time polymerase chain reaction

Total RNA was isolated using TRIzol reagent (Thermo Fisher, Shanghai, China) and reverse-transcribed into cDNA using an RT kit (Takara, Japan). qRT-PCR was performed in triplicate using a Light Cycler 96 machine (Roche Applied Science, Basel, Switzerland) and SYBR Green Master Mix (Vazyme Biotech, Nanjing, China). Primers used are listed in **Supplementary Table S2**. Fold changes in the expression of each gene were calculated by the comparative threshold cycle (Ct) method using the formula 2^−(ΔΔCt)^, as previously described (21).

### Immunohistochemistry

Sections mounted on silanized slides were dewaxed in xylene, dehydrated in ethanol, boiled in 0.01 M citrate buffer (pH 6.0) (Beyotime, Nantong, China) for 20 min in a microwave oven, and incubated with 3% hydrogen peroxide (Thermo Fisher Scientific, USA) for 5 min. The sections were then incubated in 10% normal bovine serum albumin (MedChemExpress, USA) for 5 min, followed by incubation with the primary antibody at 4°C overnight. The antibodies used are listed in **Supplementary Table S2**. The slides were then incubated with horseradish peroxidase-conjugated antibodies at room temperature for another 30 min. The samples were then visualized using DAPI, dehydrated, cleared, mounted, and photographed using a panoramic-scan digital slice scanning system (3DHISTECH). Graphs were analyzed and quantified using Image-Pro Plus software, as described previously (20).

### Statistical analysis

Statistical analysis was performed using SPSS-29 (IBM SPSS software) or GraphPad Prism (version 9.0.0 for Windows; San Diego, California, USA). For the dose–response characteristics of DEHP-induced osteoporosis, restricted cubic splines with five knots were used to flexibly model the association between phthalate exposure and BMD. Data analyses and visualizations were performed using the ggplot2 and rms packages in R version 4.0.5. Statistical significance was determined using a two-tailed Student’s t-test, one-way analysis of variance (ANOVA) followed by Dunnett’s t-test, or two-way analysis of variance followed by Sidak’s multiple comparison test. Differences were considered significant when the *p*-value was <0.05.

## Results

### Description of the exposure and dose–response relationship between DEHP and BMD in population from 2009 to 2018

**Figure 1:**
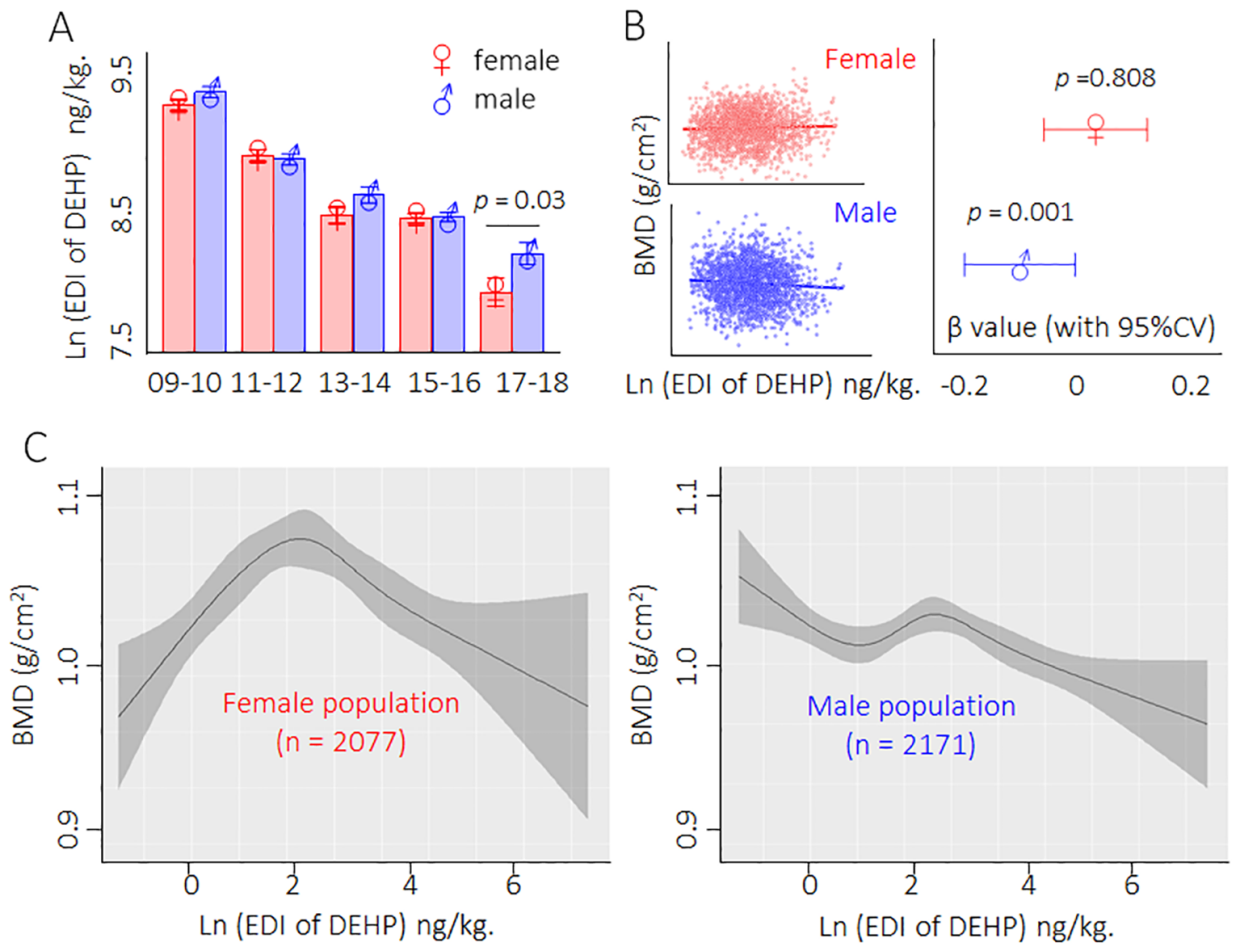
Description of the exposure and dose-response relationship between DEHP and BMD in population from 2009 to 2018. **(A)** Male/female population exposed to DEHP from 2009 to 2018. Data was shown as mean ± SD, and a two-tailed Student’s t-test was used for between two group comparison (for male and female) **(B)** Relationship between DEHP exposure and BMD. Data correlation was analyzed by regression correlation analysis. **(C)** The dose–response curves of DEHP exposure levels and BMD in male and female populations.

First, we calculated the EDI based on the concentrations of the four phthalate metabolites in the urine (**Supplementary Table S1**). Here, among the five consecutive survey cycles (2009/2010 to 2017/2018), the urinary concentrations of MEHP, MECPP, MEHHP, MEOHP, and the EDI of DEHP decreased in a time-dependent manner in both male and female populations (**Figure 1A**). We further investigated the relationship between DEHP exposure and BMD and found that only the male population exhibited a negative correlation between DEHP exposure and BMD (**Figure 1B**). Interestingly, the fitting curve also showed that the dose–response characteristics exhibited a negative correlation trend only in the male population; nevertheless, for the female population, the dose–response characteristics exhibited parabolic-like dose-dependent curves (**Figure 1C**). Collectively, these results indicated that a low dose of DEHP was associated with a reduction in BMD and that there were sex differences in such effects.

### Construction an *in vivo* model of low dose DEHP-induced HOD

**Figure 2:**
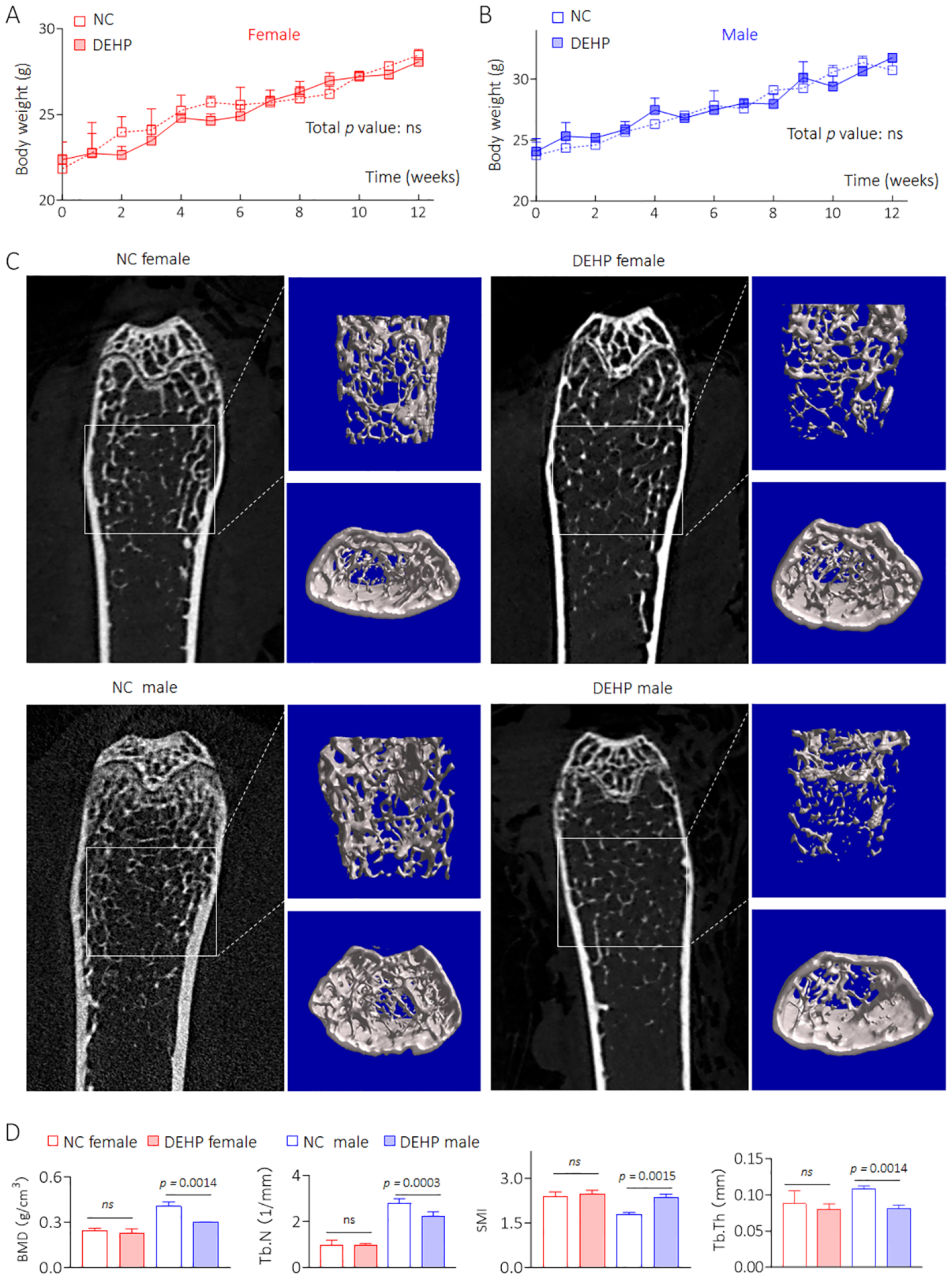
Construction an *in vivo* model of low dose DEHP induced HOD. **(A, B)** Body weight. Data was shown as mean ± SD, n = 5, and a two-way analysis of variance followed by Sidak’s multiple comparisons test were used for comparisons. **(C, D)** 2D image, 3D bone structure reconstruction, and analysis of bone density index based on Micro-CT. Data was shown as mean ± SD, n = 5, and a two-tailed Student’s t-test was used for between two group comparisons. ns, not significant.

C57BL/6J mice were divided into NC- and DEHP-treated groups for 12 weeks. As shown in **Figures 2A, B**, there was no significant difference in weight gain between male and female mice. Visualization of the skeleton through CT revealed that bone mineral density (BMD), Tb.N, SMI, and Tb.Th were not significantly different in DEHP-treated female mice. However, all of the above BMD related index indices were remarkably changed in male mice in the DEHP-treated group (**Figures 2C, D**). Collectively, these results revealed that low-dose DEHP exposure caused osteoporosis in male mice, which preliminarily validated the conclusions obtained from statistical analysis at the population level.

### Effects of DEHP on liver pathology and liver function in mice

**Figure 3:**
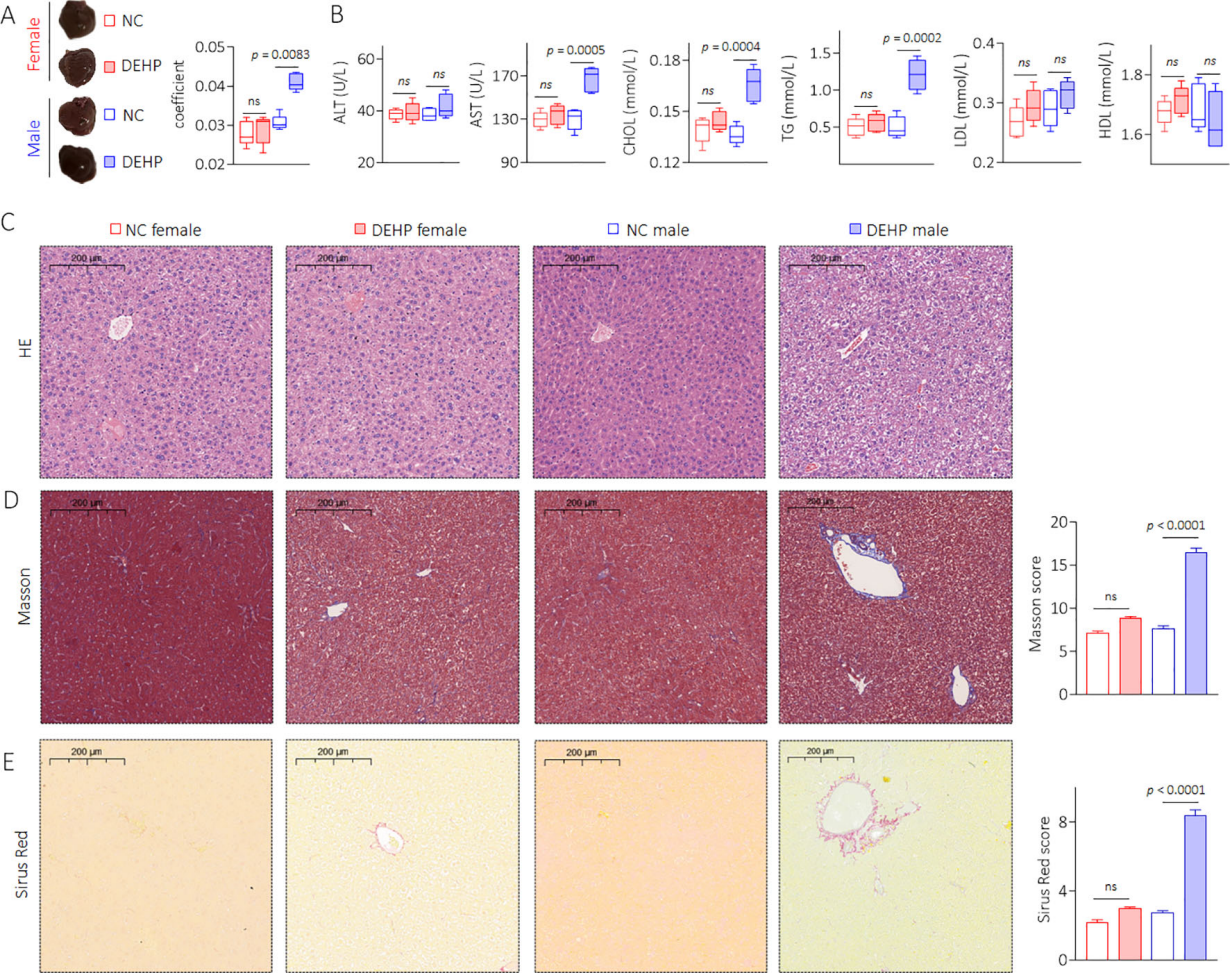
Effects of DEHP on liver pathology and liver function in mice. **(A)** Liver gross morphology and liver coefficient. **(B)** Levels of serum ALT, AST, CHOL, TG, LDL, and HDL. **(C)** Liver sections were stained with H&E. **(D, E)** Masson and Sirius Red staining and quantitative analysis of liver sections. Data was shown as mean ± SD, n = 5, and a two-tailed Student’s t-test was used for between two group comparison. ns, not significant.

Compared with female mice, DEHP-treated male mice showed a significant elevation in the liver coefficient compared to the NC group (**Figure 3A**). The serum levels of AST, CHOL, and TG were also significantly elevated in male mice but not in female mice after DEHP treatment (**Figure 3B**). In male mice, the liver tissue showed hepatocellular edema in the DEHP-treated group (**Figure 3C**). In addition, massive collagen deposition (as determined by Masson and Sirius Red staining) was observed only in DEHP-treated male mice (**Figures 3D, E**). It has been found that, compared to male mice, the bone metabolism balance in female mice is more easily disrupted (22). Indeed, in female C57BL/6 mice, relatively longer-term (29 weeks) DEHP exposure could directly promote adipogenic differentiation and suppress osteogenic differentiation of bone marrow mesenchymal stem cells (15). However, in the present study, the BMD decreases and microstructure disorder of femurs were not observed in female mice, suggesting a time-dependent effect of DEHP on bone marrow mesenchymal stem cells. Liver–bone communication plays a crucial role in the pathogenesis and development of osteoporosis (23, 24). Only DEHP-treated male C57BL/6J mice exhibited both liver damage and osteoporosis. Therefore, we speculated that low-dose DEHP exposure caused liver damage and dysfunction, which might lead to or at least be involved in DEHP-induced osteoporosis in male mice.

### DEHP activated a 14-3-3η/NF-κB feedback loop in male mice

**Figure 4:**
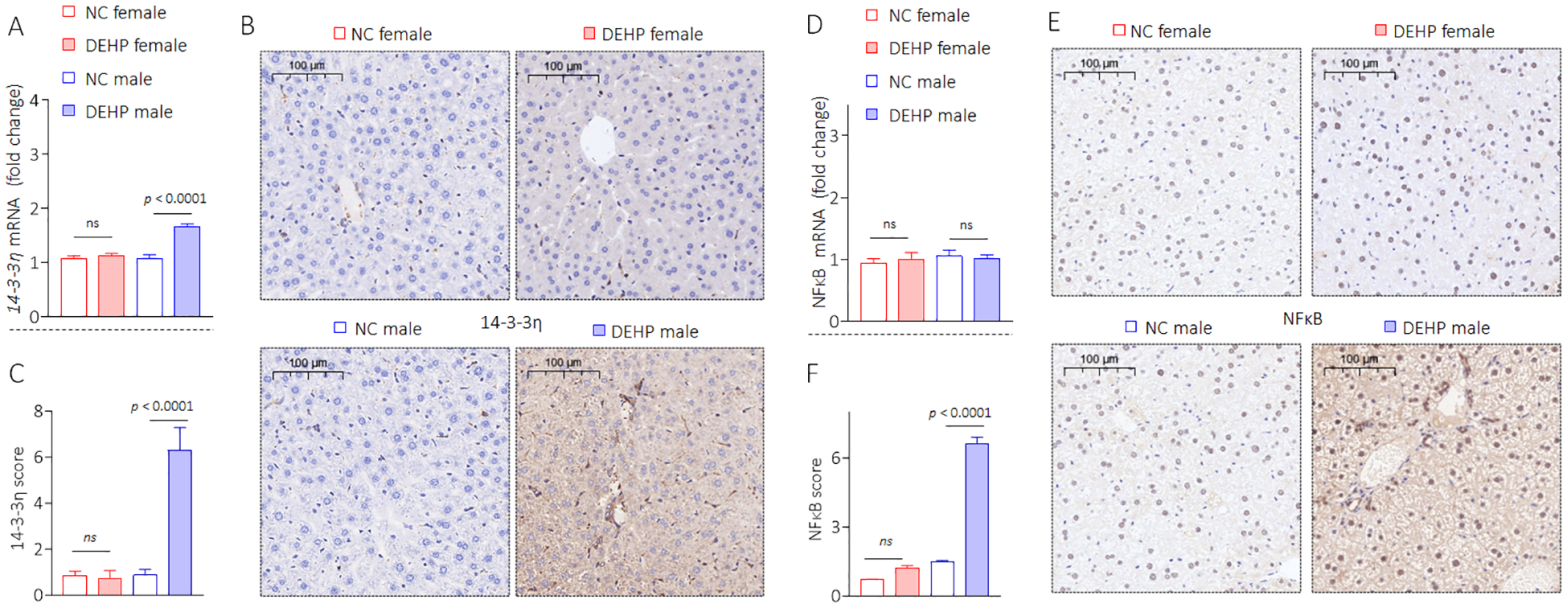
DEHP activated a 14-3-3η/NF-κB feedback loop in male mice. **(A)** Triplicate qPCR and **(B, C)** IHC analysis of 14-3-3η expression in liver sections. **(D)** Triplicate qPCR and **(E, F)** IHC analysis of NF-κB expression in liver sections. Data was shown as mean ± SD, n = 5, and a two-tailed Student’s t-test was used for between two group comparisons. ns, not significant.

Our research group had found through long-term studies that 14-3-3η was a “switch-like” factor in the development and progression of chronic hepatic disease and hepatocellular carcinoma (21, 25, 26). Moreover, our recent study revealed that 14-3-3η plays a key role in the initiation and development of hepatic fibrosis by modulating the secretory proteome (20). However, the functions and mechanisms of 14-3-3η in DEHP-induced liver lesions, functional disorders, and osteoporosis remain largely unknown. Therefore, we first determined the effects of DEHP on the expression of 14-3-3η. Interestingly, exposure to a low dose of DEHP significantly increased the expression of 14-3-3η in male mice but not in female mice (**Figures 4A–C**, **Supplementary Figure S1**). Studies have revealed that estrogen plays a key protective role in the development of various chronic liver diseases by blocking NF-κB activation (27, 28). Our previous study revealed that NF-κB signaling can transcriptionally activate 14-3-3η. Meanwhile, the activation of NF-κB can also be constitutively maintained by 14-3-3η, which is a positive feedback loop between NF-κB and 14-3-3η (29). Therefore, we hypothesized that the sex disparity in DEHP-elevated 14-3-3η was due to sex differences in NF-κB activity. Here, the expression of nuclear- or phosphorylated-NF-κB also remarkably elevated only in DEHP-treated male mice (**Figures 4D–F**, **Supplementary Figure S1**). Collectively, these results indicated that, owing to gender differences, DEHP activated a hepatic 14-3-3η/NF-κB feedback loop only in male mice. We further hypothesized that this feedback loop might in turn induce HOD by modifying the secretory proteome associated with bone differentiation.

### DEHP modified the secretory proteome associated with bone differentiation in male mice

**Figure 5:**
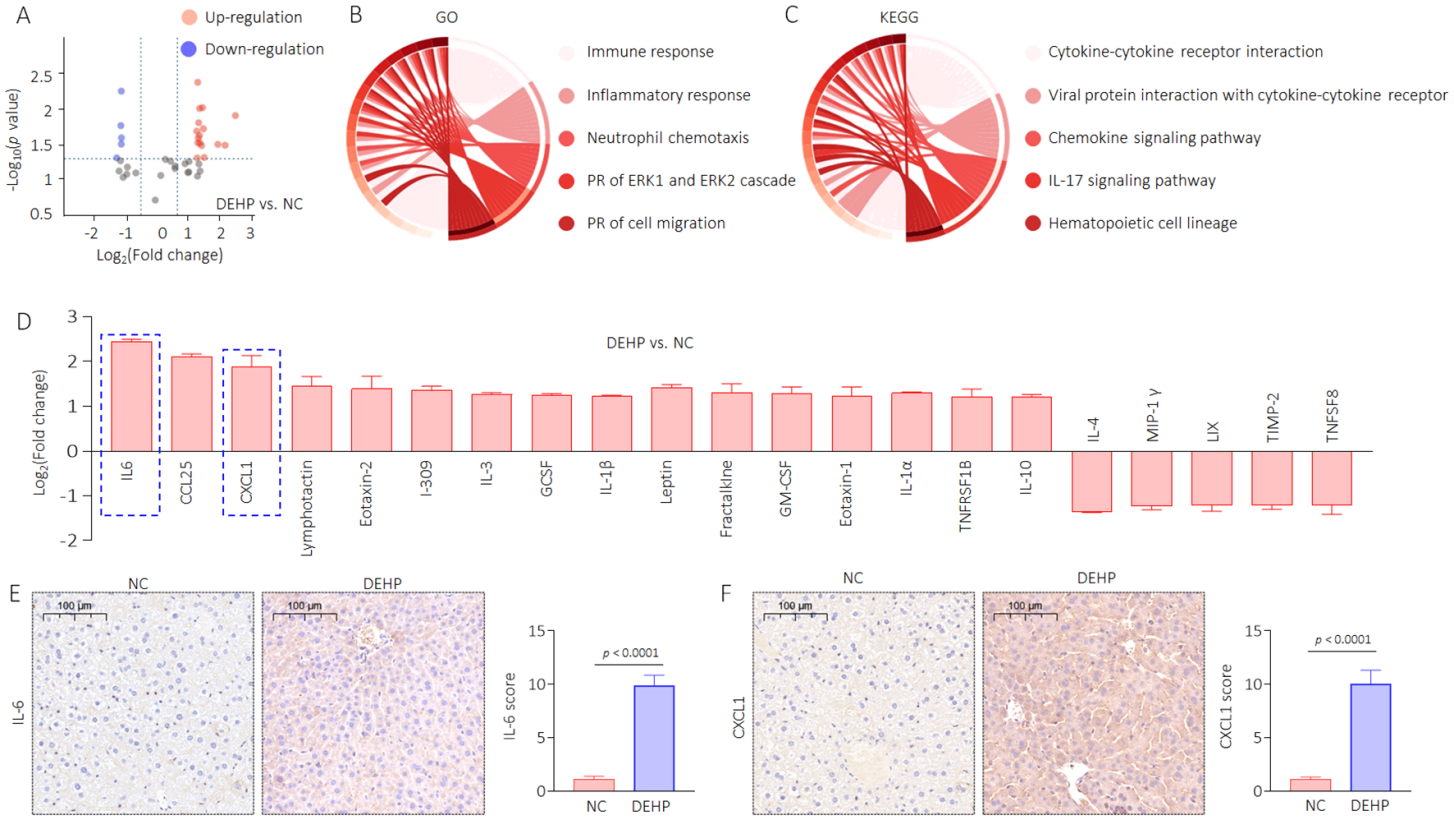
DEHP modified the secretory proteome associated with bone differentiation in male mice. **(A)** Volcanic map of the differentially expressed proteins. **(B)** GO and **(C)** KEGG analyses based on the differentially expressed proteins. **(D)** Differentially expressed proteins in DEHP vs. NC compared group. **(E, F)** IHC analysis of IL-6 and CXCL1 in liver sections (mean ± SD, n=5), and a two-tailed Student’s t-test was used for between two group comparisons.

To verify this hypothesis, we extracted liver tissue proteins and conducted high-throughput detection using the Mouse Cytokine Antibody Array. Here, a total of 21 proteins (including 16 upregulated and five downregulated) were significantly altered in the DEHP vs. NC comparison group (**Figure 5A**). Then, GO and KEGG functional enrichment analysis of the changed proteins were performed. Immune response, inflammatory response, neutrophil chemotaxis, positive regulation (PR) of ERK1 and ERK2 cascade, and PR of cell migration were the top five important biological processes, while the cytokine–cytokine receptor interaction, viral protein interaction with cytokine and cytokine receptor, chemokine signaling pathway, IL-17 signaling pathway, and hematopoietic cell lineage were the top five important pathways (**Figures 5B, C**). We further revealed potential key secretory factors that possibly induce HOD. Via searching relevant literature, we chose the interleukin-6 (IL-6) and C-X-C motif chemokine ligand 1 (CXCL1, listed as the top five changed proteins) for further investigation. IL-6 and CXCL1 are two important factors transcriptional regulated by NF-κB (30, 31). The signal intensities of IL-6 and CXCL1 were significantly higher in the DEHP-treated group (**Figure 5D**). Our data also confirmed that compared with NC, DEHP treatment markedly elevated the expression of IL-6 and CXCL1 (**Figures 5E, F**, **Supplementary Figure S1**). These findings suggest that DEHP may improve the expression of IL-6 and CXCL1 via the 14-3-3η/NF-κB feedback loop. Based on the abovementioned findings and on the fact that IL-6 and CXCL1 play important roles in inflammatory liver injury and in inducing osteoporosis (32, 33), we further hypothesized that targeted inhibition of the 14-3-3η/NF-κB feedback loop could prevent DEHP-induced liver lesions, functional disorders, and HOD.

### Targeted inhibition of 14-3-3η by imICA prevented DEHP-modified secretion of IL-6 and CXCL1, and reversed the DEHP-caused liver lesions, functional disorders, and HOD in male mice

**Figure 6:**
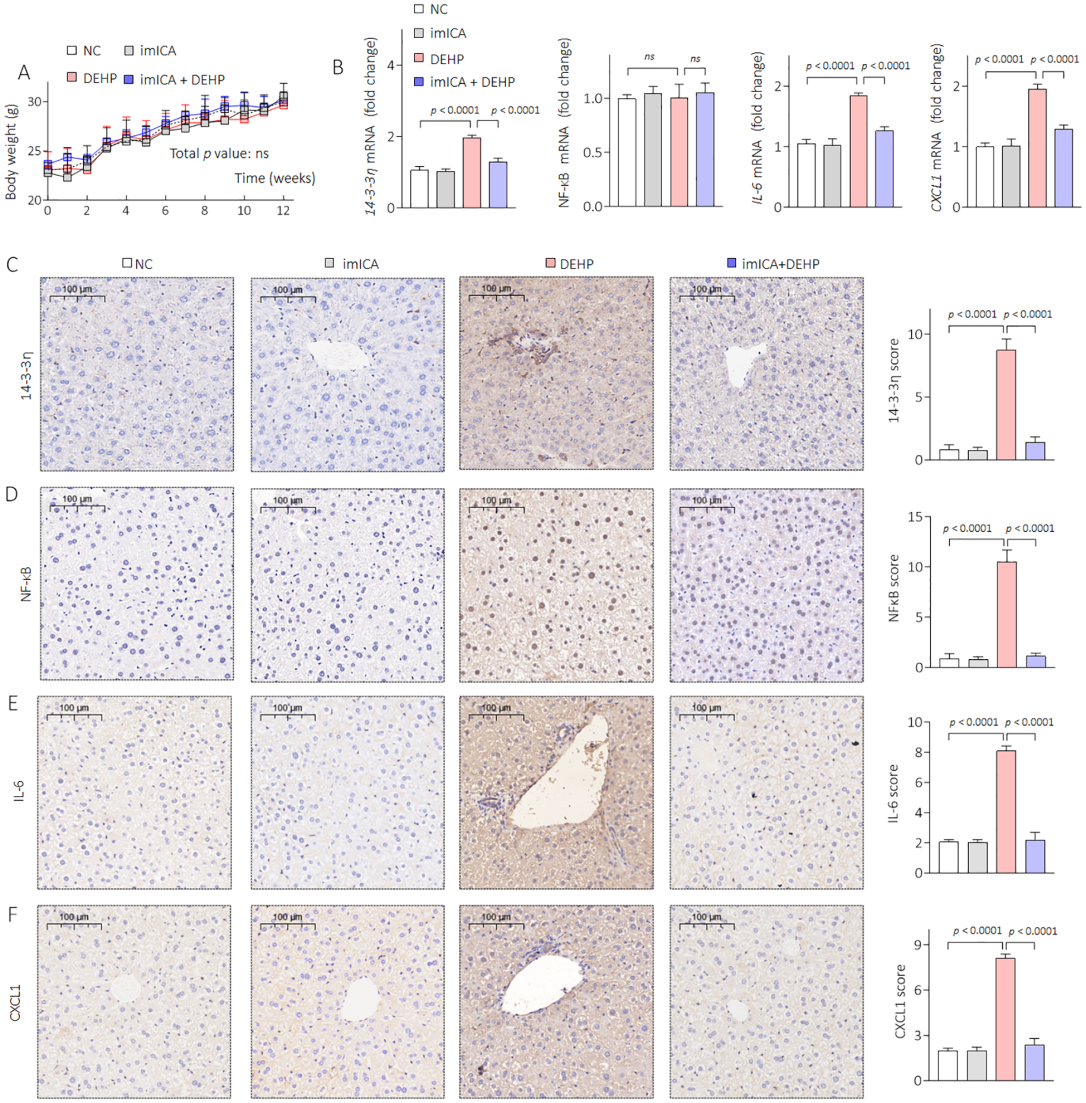
Effects of DEHP and/or imICA on 14-3-3η, NF-κB, IL-6, and CXCL2 in male mice. **(A)** Body weight. Data was shown as mean ± SD, n = 5, and a two-way analysis of variance followed by Sidak’s multiple comparisons test were used for comparisons. **(B)** Triplicate qPCR analysis of 14-3-3η, NF-κB, IL-6, and CXCL1 in liver sections. **(C–F)** IHC analysis of 14-3-3η, NF-κB, IL-6, and CXCL1 in liver sections. Data was shown as mean ± SD, n = 5, and an ANOVA followed by Dunnett’s t-test was used for comparisons. ns, not significant.

**Figure 7:**
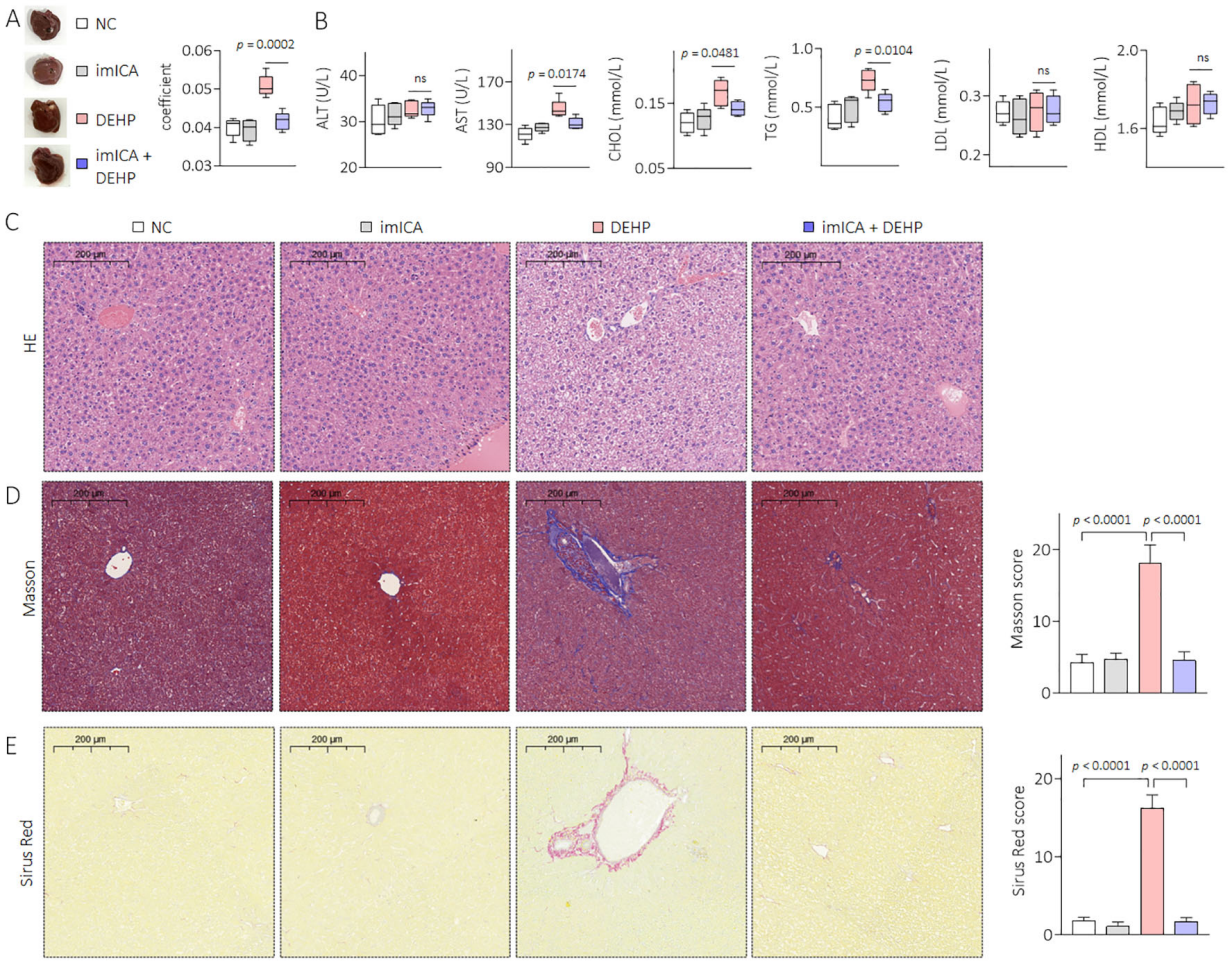
Effects of DEHP and/or imICA on liver pathology and liver function in male mice. **(A)** Liver gross morphology and liver coefficient. **(B)** Levels of serum ALT, AST, CHOL, TG, LDL, and HDL. **(C)** Liver sections were stained with H&E. **(D, E)** Masson and Sirius Red staining and quantitative analysis of liver sections. Data was shown as mean ± SD, n = 5, and an ANOVA followed by Dunnett’s t test was used for comparisons. ns, not significant.

**Figure 8:**
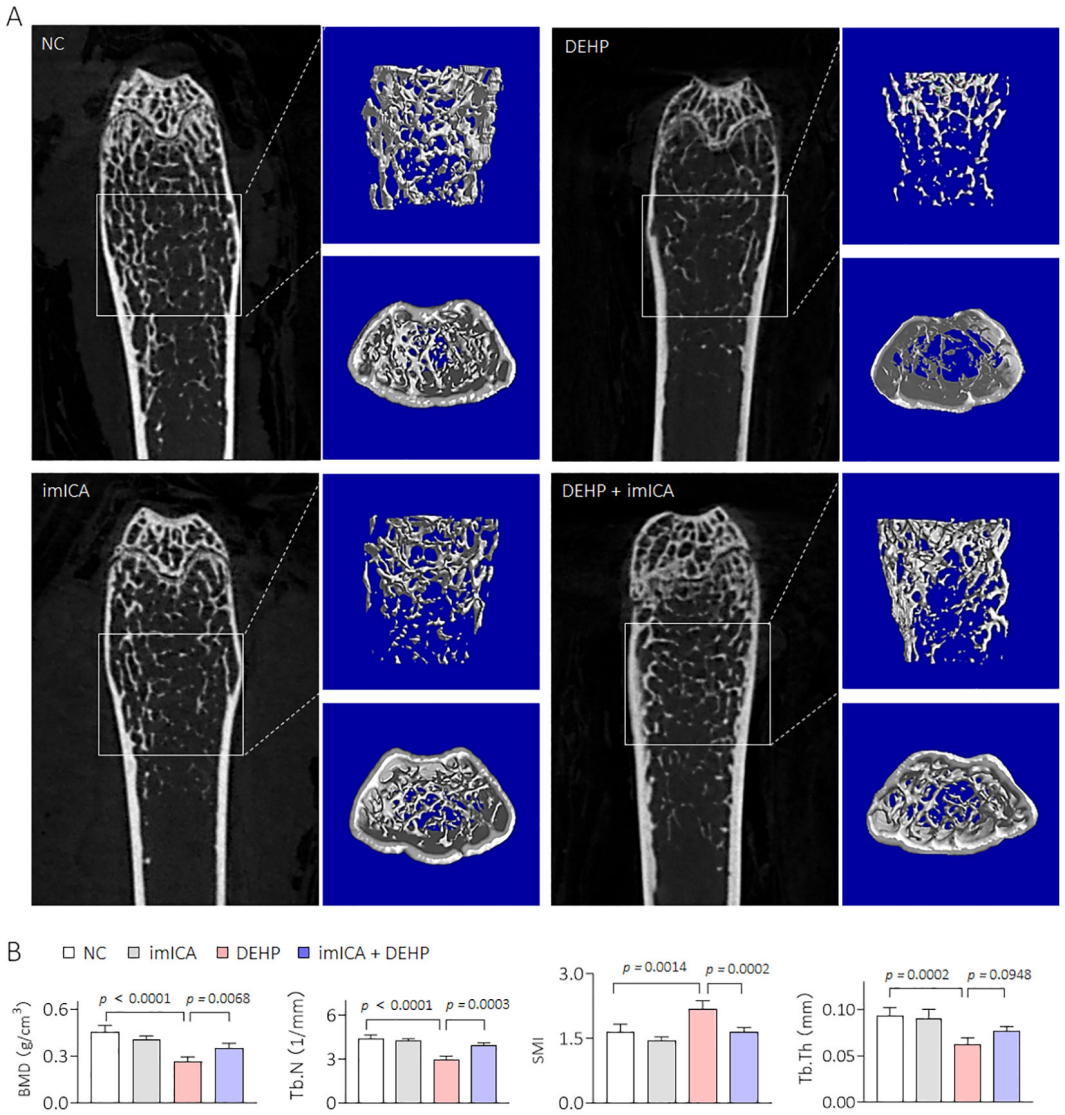
Effects of DEHP and/or imICA on BMD, Tb.N, SMI, and Tb.Th in male mice. **(A, B)** 2D image, 3D bone structure reconstruction, and analysis of bone density index based on Micro-CT. Data was shown as mean ± SD, n = 5, and an ANOVA followed by Dunnett’s t test was used for comparisons.

Our previous study revealed that high expression of 14-3-3 protein is necessary for sustained activation of NF-κB (34). Therefore, targeted inhibition of 14-3-3η is an effective approach to block the 14-3-3η/NF-κB feedback loop. Chemical imICA was modified and synthesized in our latest study. This chemical exhibited an excellent targeted inhibitory effect on the 14-3-3η protein (19). Here, male C57BL/6J mice were treated by 0.0 mg/kg or 0.5 mg/kg DEHP in the presence or absence of 0.0 mg/kg or 5.0 mg/kg imICA for 12 weeks. As shown in **Figure 6A**, there was no significant difference in the weight gain between the groups. However, imICA treatment markedly decreased the expression of 14-3-3η, nuclear NF-κB, IL-6, and CXCL1 (**Figures 6B–F**). Compared with the NC group, DEHP significantly elevated the liver coefficient and the serum levels of AST, CHOL, and TG; however, these effects were attenuated by treatment with imICA (**Figures 7A, B**). Meanwhile, imICA also markedly blocked DEHP-induced hepatocellular edema, steatosis, and depositions of lipids and collagen (**Figures 7C–E**). Finally, by visualizing the skeleton through CT, we found that imICA reversed DEHP-induced osteoporosis (**Figure 8**). Collectively, these results indicate that targeted inhibition of 14-3-3η/NF-κB by imICA prevented DEHP-elevated IL-6 and CXCL1, leading to the reversal of DEHP-induced HOD in male mice.

## Discussion

Di-(2-ethylhexyl) phthalate (DEHP) is a common plasticizer widely used in many consumer products including toys, food wrapping, and medical devices (35). People of all age groups may be exposed to DEHP in various ways, including dermal contact, ingestion, inhalation, and medical injection (36). The level of DEHP exposure in different populations is influenced by factors such as age, sex, occupation, and lifestyle. For the general population, the estimated daily exposure to DEHP through non-dietary routes is between 3 μg/kg/day and 30 μg/kg/day (37). Children tend to have higher concentrations of DEHP than adults because of higher food intake and frequent hand-to-mouth contact (38). Pregnant women and newborns are particularly sensitive to DEHP, and low-dose exposure during pregnancy may result in neurodevelopmental disorders and reproductive abnormalities in the offspring (39). Occupationally exposed populations have significantly higher exposure levels than the general population owing to long-term exposure to DEHP-containing materials (40). Long-term exposure to DEHP can cause damage to multiple organs. Long-term intake of DEHP has potential effects on human myocardial contractility, and its metabolite MEHP leads to negative inotropic effects on the human myocardium, which may have toxic effects on the human heart. At the same time, infants with multiple system failure will be the group with the greatest risk of MEHP cardiotoxicity (41). In addition, long-term exposure to DEHP can lead to the development of obesity, lipid metabolism disorders, insulin resistance and type 2 diabetes (42, 43). Therefore, DEHP contamination is a major public health concern.

This study utilized the NHANES database to gather data on DEHP metabolites from 2009 to 2018, enabling the calculation of the EDI of DEHP and taking 95% of the total population every two years for statistical analysis (44, 45). The average EDI of DEHP was 5,000 ng/kg from the NHANES database in 2017–2018, based on the equivalent dose ratios for humans and mice according to body surface area, then applying a 10-fold safety factor, we finally deduced a dose of 0.5 mg/kg in mice. Therefore, we selected 0.5 mg/kg DEHP for long-term treatment of the mice.

Studies have indicated the existence of sex-based differences in skeletal phenotypes; females exhibited lower bone mass and higher osteoclast counts, and the kinetics of *in vitro* osteoclastogenesis were also faster in females (46). Previous research has demonstrated that DEHP exerts a detrimental effect on bone health by impeding osteogenic differentiation of bone marrow mesenchymal stem cells (15). In the present study, micro-CT analysis indicated that oral exposure of female C57BL/6J mice to DEHP (0.5 mg/kg) for 12 weeks did not result in pathological changes associated with osteoporosis. However, the same dose of DEHP led to a reduction in BMD, significant bone trabecular loss, and structural damage in male mice. Therefore, we hypothesized that there is a specific pathway for osteoporosis induced by low doses of DEHP.

Almost all patients with chronic liver disease experience changes in their bone metabolism. Liver–bone communication plays a crucial role in the pathogenesis and development of primary osteoporosis (23, 24). It has been well documented that there are multiple regulatory mechanisms that maintain equilibrium between the liver and bone. When liver damage occurs, bone metabolism becomes abnormal owing to an imbalance of osteoblasts and osteoclasts (1). After injecting CCl_4_ into mice to induce liver injury, the synthesis of 25-OH vitamin D produced in the liver decreased while TGF β increased, leading to a decrease in BMD (47). The liver is an important organ for metabolism and one of the primary target organs for the toxic effects of DEHP (48). Epidemiological investigations have demonstrated a significant correlation between urinary DEHP levels and indicators of liver injury (49). DEHP can inhibit liver detoxification enzymes, leading to liver dysfunction and accelerating the progression of chronic liver injury (50). In the present study, a low dose of DEHP caused liver lesions and disrupted liver function, which might lead to HOD only in male mice, indicating a sex difference in such effects.

Estrogen has been shown to slow the onset of all types of chronic liver disease by inhibiting the transformation of quiescent hepatic stellate cells to myofibroblasts in injured livers by reducing lipid peroxidation, tissue inhibitor of metalloproteinases-1, and deposition of type I and type III protofibrillar-forming collagens (51). This may be a significant factor contributing to the absence of osteoporosis symptoms in female mice following DEHP treatment. Therefore, it could be concluded that estrogen deficiency exacerbated the development of HOD in men.

14-3-3 proteins are a family of phosphoserine/threonine regulatory proteins that are involved in promoting the progression of several biological processes (52, 53). In the past few years, we proposed that the 14-3-3η isoform is a key characteristic neoplastic factor in HCC (25). Through the phosphorylated-modification of the proteome, 14-3-3η dominated the growth, angiogenesis, cancer stem cell-like properties, multi-drug resistance, and several other decisive processes (21, 34). The NF-κB canonical pathway has been shown to play a role in a variety of diseases by regulating inflammation, apoptosis, and other physiological processes. Increasing evidence has shown that the NF-κB canonical pathway is involved in the pathogenesis of liver disease via transcriptional regulation of a variety of target genes, such as interleukin 6 (IL-6), or by activating additional cell signaling pathways, such as signal transducer and activator of transcription 3 (STAT3) (54, 55). Our previous study also confirmed that the NF-κB/IL-6/STAT3 positive feedback loop plays a crucial role in inflammation and pro-survival and might be an essential link between inflammation and cancer (31). Our previous study revealed a positive feedback loop between NF-κB and 14-3-3η (29) and that 14-3-3η modulates the secretory proteome in human liver cell lines (20). Collectively, these findings indicate that the 14-3-3η/NF-κB positive feedback loop-mediated hepatic paracrine effect might be involved in DEHP-induced HOD.

Epidemiological investigations have demonstrated a positive correlation between the expression of IL-6-related genes and the development of osteoporosis (56). Additionally, elevated IL-6 levels in ovariectomized female mice resulted in the enhanced differentiation of bone marrow cells into osteoclasts. Conversely, IL-6 deficiency preserves bone mass and prevents alterations in bone turnover (57, 58). Similarly, reports have demonstrated a correlation between elevated serum levels of CXCL1 and reduced BMD, indicating a negative correlation between CXCL1 and bone mass (59). Indeed, excess CXCL1 derived from bone marrow adipocytes promotes osteoclast maturation and accelerates skeletal osteolysis (60). Importantly, CXCL1 is an important transcription factor regulated by NF-κB (30, 31). Collectively, these findings suggest that DEHP might improve the hepatic levels of IL-6 and CXCL1 via the 14-3-3η/NF-κB feedback loop, leading to HOD induction.

The chemical imICA was modified and synthesized in our latest study. Based on the serum biochemical test results for the liver and kidneys, we observed no significant differences in serum ALT, AST, blood urea nitrogen (BUN), and CREA levels between the imICA-treated group and the control group. This indicated that imICA had relatively minor hepatotoxic and nephrotoxic effects in the mice (**Figure 7B**, **Supplementary Figure S2**). In addition, it exhibited an excellent targeted inhibitory effect on 14-3-3η protein as well as its downstream signal transduction (19). ImICA significantly inhibits the development and progression of hepatic fibrosis and hepatocellular carcinoma mediated by 14-3-3η overexpression by targeting the regulation of the 14-3-3η protein-signaling pathway (19, 20). The present study revealed that imICA could also block DEHP-induced liver lesions, functional disorders, and HOD in male mice.

## Conclusions

**Figure 9:**
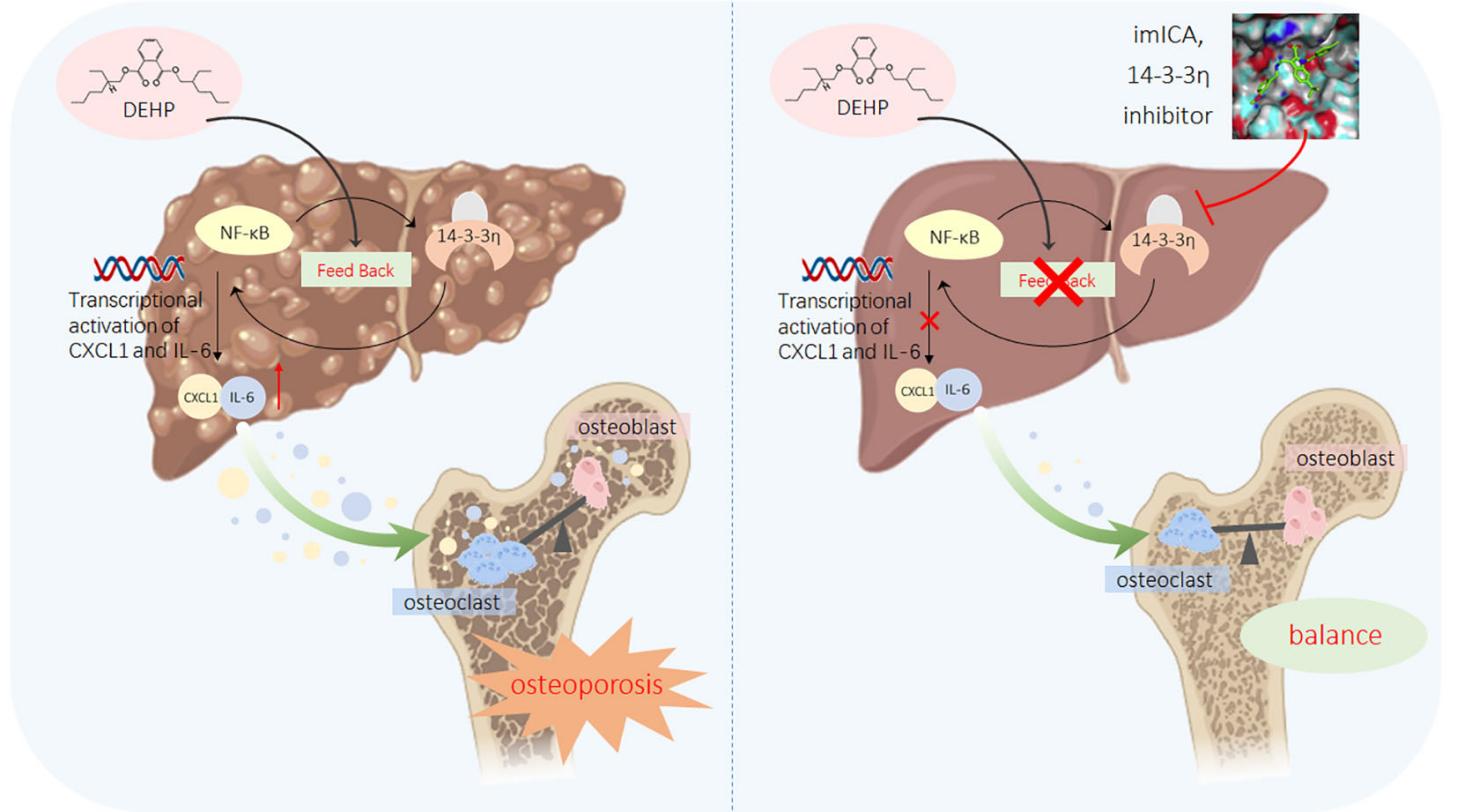
A sketch map summarizing the conclusions, innovations, and potential preventive significance of our present study.

In recent years, male and female populations have been exposed to a relatively low concentration of DEHP, but only the male population exhibited a negative correlation between DEHP exposure and BMD. In male mice, a low dose of DEHP activated the hepatic 14-3-3η/NF-κB positive feedback loop, which in turn modified the secretory proteome associated with bone differentiation and elevated IL-6 and CXCL1 levels, leading to HOD. Targeted 14-3-3η/NF-κB feedback loop using our novel 14-3-3η inhibitor, imICA, prevented DEHP-induced HOD (**Figure 9**).

## Data Availability

The original contributions presented in the study are included in the article/**Supplementary Material**. Further inquiries can be directed to the corresponding authors.
