# Supplementary material for: Mechanisms and targeted prevention of hepatic osteodystrophy caused by a low concentration of di-(2-ethylhexyl)-phthalate
Source: Front Immunol. 2025 Mar 10;16:1552150. doi: 10.3389/fimmu.2025.1552150 (PMC11931061; doi:10.3389/fimmu.2025.1552150)
Supplement: Supplementary file 1 [file DataSheet1.docx]

**Mechanisms and Targeted Prevention of Hepatic Osteodystrophy Caused by a Low Concentration of Di-(2-ethylhexyl)-phthalate**

Qinming Hui ^1, 2, *^, Xinru Du ^1, 2, *^, Maoxuan Li ^2^, Sha Liu ^2^, Zhendong Wang ^2^, Sisi Song ^2^, Yancheng Gao ^2^, Ye Yang ^2, 🖂^, Chunxiao Zhou ^1, 🖂^, and Yuan Li ^1, 2, 🖂^

1. Department of Gastroenterology, The Affiliated Suzhou Hospital of Nanjing Medical University, Suzhou, Jiangsu Province, China.
2. The Key Laboratory of Modern Toxicology, Ministry of Education, School of Public Health, Nanjing Medical University, Nanjing, China.

Note *: Qinming Hui and Xinru Du contributed equally to this work.

Corresponding author informations ^🖂^: Dr. Yuan Li, E-mail: liyuan@njmu.edu.cn; or Dr. Chunxiao Zhou, E-mail: zhouchunxiao@njmu.edu.cn; or Dr. Ye Yang, Email: [yangye@njmu.edu.cn](mailto:yangye@njmu.edu.cn).

**Table. S1. The *in vivo* concentrations of the four metabolites of DEHP from 2009 to 2018.**

|  |  | 2009-2010 | | 2011-2012 | | 2013-2014 | | 2015-2016 | | 2017-2018 | |
| --- | --- | --- | --- | --- | --- | --- | --- | --- | --- | --- | --- |
|  |  | Mean ± SD | *p* value | Mean ± SD | *p* value | Mean ± SD | *p* value | Mean ± SD | *p* value | Mean ± SD | *p* value |
|  | ALL | 3.441 ± 5.622 |  | 2.823 ± 3.637 |  | 1.9 59 ± 2.082 |  | 1.898 ± 2.003 |  | 1.130 ± 0.917 |  |
| MEHP | ♂ | 3.789 ± 5.636 | 0.017 | 2.908 ± 3.794 | 0.417 | 2.119 ± 2.210 | 0.121 | 2.006 ± 2.102 | 0.067 | 1.188 ± 0.966 | 0.390 |
|  | ♀ | 3.070 ± 5.588 |  | 2.728 ± 3.455 |  | 1.818 ± 1.957 |  | 1.788 ± 1.893 |  | 1.067 ± 0.863 |  |
| MECPP | ALL | 34.650 ± 41.600 |  | 20.452 ± 20.258 |  | 13.406 ± 11.561 |  | 12.104 ± 10.241 |  | 9.143 ± 8.651 |  |
|  | ♂ | 36.871 ± 45.172 | 0.040 | 20.998 ± 21.689 | 0.348 | 14.363 ± 12.086 | 0.096 | 12.355 ± 10.319 | 0.403 | 9.737 ± 8.116 | 0.347 |
|  | ♀ | 32.282 ± 37.309 |  | 19.843 ± 18.536 |  | 12.569 ± 11.039 |  | 11.846 ± 10.164 |  | 8.492 ± 9.209 |  |
| MEHHP | ALL | 23.393 ± 31.600 |  | 13.253 ± 13.653 |  | 9.259 ± 8.472 |  | 8.013 ± 6.976 |  | 5.685 ± 5.575 |  |
|  | ♂ | 25.976 ± 34.970 | 0.002 | 13.817 ± 14.794 | 0.150 | 10.031 ± 8.750 | 0.067 | 8.307 ± 6.891 | 0.150 | 6.412 ± 5.830 | 0.073 |
|  | ♀ | 20.642 ± 27.316 |  | 12.625 ± 12.241 |  | 8.584 ± 8.180 |  | 7.711 ± 7.056 |  | 4.886 ± 5.200 |  |
| MEOHP | ALL | 14.017 ± 17.341 |  | 8.377 ± 8.411 |  | 5.706 ± 5.004 |  | 4.999 ± 4.308 |  | 3.728 ± 3.680 |  |
|  | ♂ | 14.952 ± 18.380 | 0.038 | 8.438 ± 8.826 | 0.799 | 6.116 ± 5.092 | 0.099 | 5.003 ± 4.241 | 0.976 | 4.153 ±3.833 | 0.112 |
|  | ♀ | 13.021 ± 16.113 |  | 8.308 ± 7.932 |  | 5.346 ± 4.909 |  | 4.995 ± 4.380 |  | 3.261 ± 3.468 |  |

**Table. S2. Primers used in this study.**

| Genes | Primers |
| --- | --- |
| *14-3-3η* | F: 5’-CCTGCCTCTTAGCCAAAC-3’  R: 5’-CTCCTGCTTCTTCATCCTG-3’ |
| *IL-6* | F: 5’-CAGCCACTCACCTCTTCA-3’  R: 5’-CACTGTCTTTGAGCCTGTC-3’ |
| *CXCL1* | F: 5’-CGCTCAGTCAGTGAGTCTCTT-3’  R: 5’-GGGGGACTTCACGTTCACA-3’ |
| *Actin* | F: 5’-GACCTGACCTGCCGTCTA-3’  R: 5’-GGAGTGGGTGTC GCTGT-3’ |

**Table. S3. Antibodies used in this study.**

| Antibodies | Source | No. | Dilution |
| --- | --- | --- | --- |
| 14-3-3η | Thermo Fisher | PA5-75298 | 1: 100 (IHC)  1:1000 (WB) |
| IL-6 | Santa Cruz | sc-130326 | 1: 100 (IHC)  1:1000 (WB) |
| CXCL1 | Santa Cruz | sc-80516 | 1: 100 (IHC)  1:1000 (WB) |
| NF-κB/p65 | Santa Cruz | sc-515045 | 1: 50 (WB) |
| p-NF-κB | Cell Signaling Technology | 3033 | 1: 1000 (WB) |
| Actin | Cell Signaling Technology | 4970 | 1: 1000 (WB) |

**SUPPLEMENTARY FIGURES**


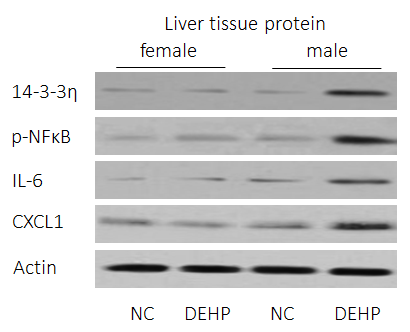


**Supplementary Figure. 1.** Western Blot related to the 14-3-3η/NF-κB pathway and IL-6/CXCL1.

**
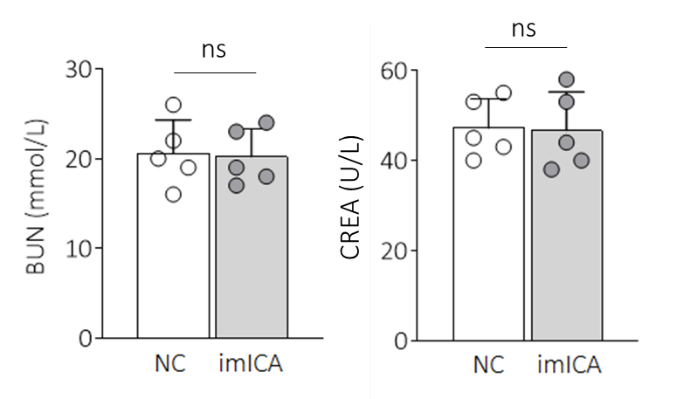
**

**Supplementary Figure. 2.** Levels of serum BUN and CREA in NC and imICA group. Data was shown as mean ± SD, n = 5, and a two-tailed Student's t-test was used for between two group comparisons. ns, not significant
